# Activation of Neural and Pluripotent Stem Cell Signatures Correlates with Increased Malignancy in Human Glioma

**DOI:** 10.1371/journal.pone.0018454

**Published:** 2011-03-31

**Authors:** Johan Holmberg, Xiaobing He, Inti Peredo, Abiel Orrego, Göran Hesselager, Christer Ericsson, Outi Hovatta, Sueli Mieko Oba-Shinjo, Suely Kazue Nagahashi Marie, Monica Nistér, Jonas Muhr

**Affiliations:** 1 Ludwig Institute for Cancer Research, Karolinska Institutet, Stockholm, Sweden; 2 Department of Cell and Molecular Biology, Karolinska Institutet, Stockholm, Sweden; 3 Department of Oncology-Pathology, Karolinska Institutet, CCK R8:05, Karolinska University Hospital Solna, Stockholm, Sweden; 4 Department of Clinical Neuroscience, Neurosurgery, Karolinska Institutet, Karolinska University Hospital Solna, Stockholm, Sweden; 5 Department of Neuroscience, Neurosurgery, Uppsala University, University Hospital, Uppsala, Sweden; 6 Department of Clinical Science, Intervention and Technology, Karolinska Institutet, Karolinska University Hospital Huddinge, Stockholm, Sweden; 7 Department of Neurology, School of Medicine, University of Sao Paulo, São Paulo, Brazil; City of Hope National Medical Center and Beckman Research Institute, United States of America

## Abstract

The presence of stem cell characteristics in glioma cells raises the possibility that mechanisms promoting the maintenance and self-renewal of tissue specific stem cells have a similar function in tumor cells. Here we characterized human gliomas of various malignancy grades for the expression of stem cell regulatory proteins. We show that cells in high grade glioma co-express an array of markers defining neural stem cells (NSCs) and that these proteins can fulfill similar functions in tumor cells as in NSCs. However, in contrast to NSCs glioma cells co-express neural proteins together with pluripotent stem cell markers, including the transcription factors Oct4, Sox2, Nanog and Klf4. In line with this finding, in high grade gliomas mesodermal- and endodermal-specific transcription factors were detected together with neural proteins, a combination of lineage markers not normally present in the central nervous system. Persistent presence of pluripotent stem cell traits could only be detected in solid tumors, and observations based on *in vitro* studies and xenograft transplantations in mice imply that this presence is dependent on the combined activity of intrinsic and extrinsic regulatory cues. Together these results demonstrate a general deregulated expression of neural and pluripotent stem cell traits in malignant human gliomas, and indicate that stem cell regulatory factors may provide significant targets for therapeutic strategies.

## Introduction

The identification of tumor cells with stem cell properties supports the idea that a subpopulation of cancer cells is responsible for the initiation, growth and recurrence of tumors. Apparent similarities with non-transformed stem cells, including high self-renewal capacity and the ability to generate differentiated progeny of several cellular lineages, have lead to the proposal that stem cell-like cancer cells may either originate from adult undifferentiated stem and progenitor cells or that these properties are being expressed as an effect of the genetic alterations which drive tumorigenicity [1]. Regardless, the association of stem cell traits with cancer pathogenesis motivates a further characterization of stem cell related signatures in tumors and a better description of molecular similarities and differences in comparison with non-transformed stem cells.

Glioma is the most common form of primary tumors of the central nervous system (CNS) in adults [1]. Based on histopathological traits this type of tumor can be divided into four malignancy grades (grade I-IV, World Health Organization) where grade IV tumors, glioblastoma multiforme, are the most malignant with no curative measures available [1], [2], [3]. Several reports have demonstrated that gliomas harbor cells with stem cell-like features, including the ability to generate progeny of the neural and glial lineages, as well as, to mediate the recurrence of tumors [4]. For example, the cell-surface protein CD133 (or prominin-1), which is expressed by stem cells of the human brain [5] has also been used to enrich for stem cells in gliomas [4], [6], [7] and has been considered as a marker for cells with enhanced tumorigenicity [4], [6], [7]. The HMG-box transcription factor Sox2 and the bHLH protein Olig2 constitute additional examples of factors commonly expressed by glioma cells and stem cells of the embryonic and adult brain [8], [9], [10]. Interestingly, both Sox2 and Olig2 have been ascribed important roles in maintaining self-renewing stem cells in the CNS [10], [11] and this activity appears, at least in part, to be conserved in gliomas [8], [10], [12], [13]. For instance, loss of Sox2 function limits the self-renewing capacity [12] of human glioma cells and reduces their tumor-inducing potential when transplanted into the rodent brain [8]. Hence, transcription factors with key regulatory roles in non-transformed stem cells are both expressed and possess similar vital functions in the maintenance of lineage-related stem and progenitor cells in gliomas.

However, the gene expression profile in gliomas may not necessarily mirror its cell of origin. For example, the transcription factor Oct4, which has an important role in maintaining embryonic stem (ES) cells in a self-renewing and pluripotent state [14], is not expressed in the adult brain but can be detected in high grade gliomas [15]. Moreover, downstream targets of Oct4 have been reported to be more frequently over-expressed in high grade gliomas, compared with lower grade tumors [16]. In the adult mice misexpression of Oct4 in epithelial tissues results in dysplasia caused by a block in progenitor differentiation [17]. Thus, the reactivation of stem cell genes, such as Oct4, may contribute to the enhanced malignancy of high grade gliomas.

It is interesting that several transcriptional networks with vital functions in neural and other stem cell populations appear to be expressed in gliomas [8], [10], [16]. Their necessity for the maintenance of self-renewing stem and progenitor cells, raises the possibility that these proteins, independently of genetic alterations, may constitute relevant targets for therapeutic strategies aiming to prevent growth and recurrence of tumors [18]. However, to realize this idea we first need to characterize the combined expression of stem cell regulatory components in gliomas and understand how these proteins function to control growth and survival of cancer cells. Here we examined human gliomas for the expression of transcription factors with key roles in controlling the self-renewing undifferentiated state of ES-cells and NSCs. We show that stem cell traits normally present in NSCs are aberrantly co-expressed with pluripotent stem cell markers in high grade gliomas. Together our findings indicate that transcriptional mechanisms regulating normal stem cells may contribute to the malignancy of glioma cells.

## Results

### Conserved features between glioma and neural stem cells

**Figure 1:**
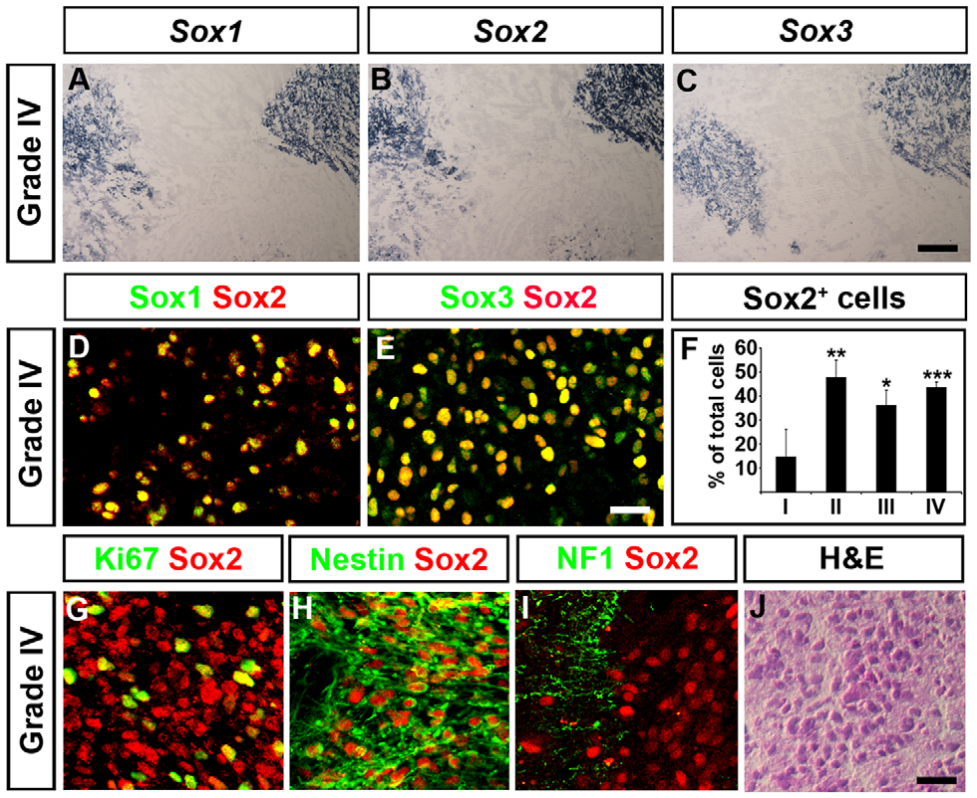
Expression of neural stem cell properties in human glioma. (A–C) Serial sections of a grade IV glioma show overlapping expression of *Sox1* (A), *Sox2* (B) and *Sox3* (C) mRNA. (D,E) Most Sox2^+^ cells co-express Sox1 (D) and Sox3 (E) proteins in grade IV tumors. (F) Gliomas of grade II-IV harbor a larger proportion of Sox2^+^ cells in comparison with the grade I tumors. (G,H) Sox2 expression overlaps extensively with the neural progenitor marker Nestin (H) and with the majority of cells expressing the cell cycle marker Ki67 (G). (I) Most cells expressing the neuronal protein Neurofilament-1 (NF1) express no or only low levels of Sox2. (J) Representative section of grade IV tumor stained with hematoxylin and eosin (H&E) (J). Data are represented as mean +/− SEM. * =  p<0.05, ** =  p<0.01, *** =  p<0.001, Student's t-test. Scale bars; 200 µm in A–C, 20 µm in E; 20 µm in J.

SoxB1 (Sox1, Sox2 and Sox3) transcription factors are co-expressed by the majority of all NSCs both in the developing and adult CNS, and constitute important regulators of NSC maintenance [9], [19]. To define molecular similarities and differences between non-transformed CNS stem cells and glioma cells, we characterized SoxB1 gene expression both at the mRNA and protein level in human gliomas of different malignancy grades. In grade IV tumors, verified by hematoxylin and eosin staining (Fig. S1), the domains of *Sox1, Sox2* and *Sox3* mRNA expression overlapped extensively (Fig. 1A–C). Moreover, most cells expressing Sox2 protein also showed immunoreactivity with antibodies specific for Sox1 or Sox3 protein (Fig. 1D,E; and Fig. S2). The proportion of cells expressing Sox2 was similar in grade II – IV tumors (Fig. 1F) in which Sox2 could be detected in approximately 40% of the cells. In pilocytic astrocytomas (Grade I) Sox2 could only be detected in less than 20% of the cells (Fig. 1F). Sox3 followed the same expression pattern as Sox2 in all tumor samples examined. However, we were unable to detect any significant expression of Sox1 mRNA or protein in grade I – III tumors (data not shown), and co-expression of Sox1-3 was mainly found in grade IV tumors. In the CNS, the expression of Sox1-3 is confined to self-renewing precursor cells, but is generally downregulated as cells exit the cell cycle and start to differentiate into neurons or glia [9], [19]. In keeping with this, Sox2 could be detected in the vast majority of cells in grade IV tumors expressing the proliferation marker Ki67 (Fig. 1G) and almost all Sox2^+^ cells co-expressed the neural progenitor marker Nestin (≥95%) (Fig. 1H). In addition, the majority of cells expressing markers for differentiated neurons, such as Neurofilament-1 (NF1) (Fig. 1I) were Sox2-negative. Thus, similar to the healthy CNS undifferentiated proliferating cells in high grade gliomas co-express Sox1-3 transcription factors.

### A negative form of Sox3 promotes glioma cells to exit the cell cycle

**Figure 2:**
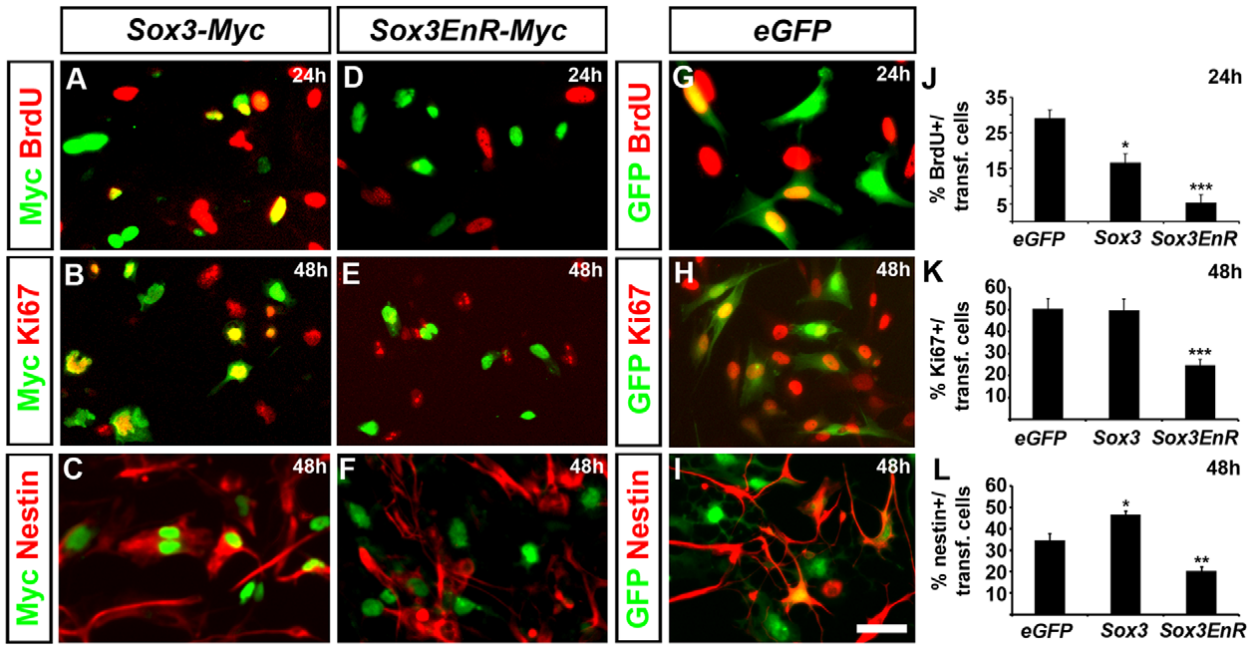
Dominant negative version of Sox3 promotes cell cycle exit. (A–C, J–L) Overexpression of *Sox3-Myc* in cells derived from grade IV tumors, maintained cells in a proliferative, BrdU incorporating (A,J), Ki67 positive (B,K) and Nestin expressing (C,L) state. (D–F, J–L) Misxpression of the dominant negative version of Sox3, *Sox3EnR-Myc*, caused cells to stop proliferating (D,E,J,K) and downregulate Nestin expression (F,L). *eGFP* misexpression did not affect the rate of proliferation (G,H,J,K) or Nestin expression (I,L). Data are represented as mean +/− SEM. * =  p<0.05, ** =  p<0.01, *** =  p<0.001, Student's t-test. Scale bar 20 µm in I.

Gain-of-function experiments have previously demonstrated that Sox1-3 maintain neural cells as self-renewing progenitors [9], [19], while dominant-negative versions of these proteins have the opposite functions and cause NSCs to exit the cell cycle and commit to differentiation [9], [19], [20]. To examine if Sox3 has a similar regulatory capacity in glioma cells as in NSCs, we transfected primary cultures derived from human grade IV gliomas with vectors expressing either full-length Sox3, a dominant negative version of Sox3 (*Sox3EnR-Myc*) [9] or enhanced GFP (*EGFP*). Many cells misexpressing a Myc-tagged version of Sox3 incorporated the thymidine analogue bromodeoxyuridine (BrdU) 24 hours after transfection (Fig. 2A,J) and expressed the cell cycle marker Ki67 (Fig. 2B,K) and the NSC markers Sox1 and Nestin 48 hours after transfection (Fig. 2C,L; and data not shown). In contrast, misexpression of *Sox3EnR-Myc* for 24 hours, promoted cells to leave the cell cycle, as indicated by the decrease in BrdU incorporation (Fig. 2D,J) and the reduction of cells positive for Ki67 (Fig 2E,K). Misexpression of *Sox3EnR-Myc* also increased the number of cells that downregulated the expression of the neural progenitor marker Nestin (Fig. 2F,L). Thus, as in the developing CNS [9] Sox3 has the capacity to maintain glioma cells in an undifferentiated and proliferating state, whereas active repression of Sox3 target genes causes cells to exit the cell cycle.

### Co-expression of Sox2, Oct4, Nanog and Klf4 in high-grade gliomas

**Figure 3:**
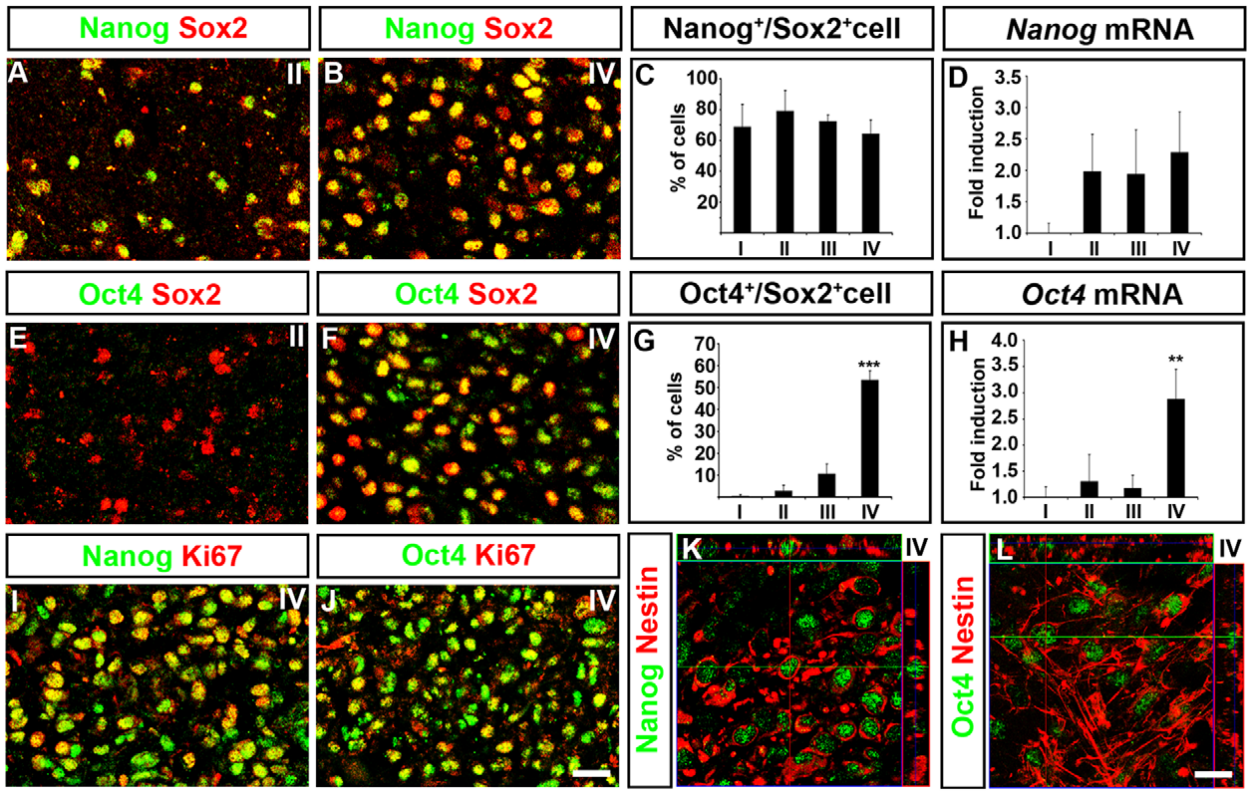
Increased malignancy is associated with the combined expression of Sox2, Oct4 and Nanog. (A–C) In tumors grade I-IV more than 60% of the Sox2^+^ cells express Nanog. (D) qPCR analyses demonstrate that *Nanog* mRNA can be detected in tumors of all grades. Values (fold higher) normalized against grade I tumors. (E–G) While Oct4 is expressed at low levels in grade I-III tumors (E,G) Oct4 is expressed in more than 50% of the Sox2^+^ cells in grade IV tumors (F,G). (H) qPCR analyses demonstrate that *Oct4* mRNA expression levels are significantly increased in grade IV tumors in comparison with lower grade tumors. Values (fold higher) normalized against grade I tumors. The majority of cells expressing Nanog and Oct4 are Ki67^+^ (I,J) and Nestin^+^ (K,L). Data are represented as mean +/− SEM. ** =  p<0.01, *** =  p<0.001, Student's t-test. Scale bar 25 µm in J and 10 µm in L.

Examination of publicly available gene expression data sets indicate that high grade human gliomas and ES-cells share similarities at the molecular level [16]. Several of the features associated with ES-cells, such as pluripotency, extensive self-renewing capacity and their gene expression profile, have been ascribed a transcriptional unit consisting of Sox2, Oct4 and Nanog [21]. Interestingly, significant levels of *Nanog* and *Oct4* mRNA could be detected in grade IV tumors (Fig. 3D,H). Using specific antibodies against Sox2, Oct4 and Nanog (Fig. S3) we found an extensive overlap in their expression pattern and many grade IV tumor cells (Fig. S4) expressing Nanog or Oct4 protein co-expressed Sox2 (Fig. 3B,F). The combinatorial expression of Sox2, Nanog and Oct4 increased with increasing grades of malignancy. While the proportion of Nanog^+^/Sox2^+^ cells was similar in a grade II and a grade IV tumor (Fig. 3A–C), the amount of Sox2^+^ cells expressing Oct4 was increased from below 5% in grade II gliomas to above 50% in grade IV tumors (Fig. 3E–G). Most Nanog^+^ and Oct4^+^ cells expressed the cell cycle marker Ki67 (Fig. 3I,J) and the NSC markers Nestin and Sox1 (Fig. 3K,L; and data not shown). Moreover, the transcription factor Klf4, which has been shown to cooperate with Sox2 and Oct4 in target gene activation in pluripotent cells [22] had similar expression pattern as Oct4 and was highly enriched in grade IV tumors (Fig. S5). Thus, undifferentiated and self-renewing cells in high grade gliomas express a combination of markers normally present in NSCs and pluripotent stem cells. Teratomas have previously been shown to express markers representative of pluripotent stem cells [23]. However, although we could detect a similar overlap in the expression pattern of Sox2, Oct4 and Nanog in ES cell-derived teratomas as in glioblastomas (Fig. S6), teratoma cells did not express pluripotent stem cell markers in combination with NSC proteins such as Sox1 (Fig. S6). To exclude a possible contribution of non-transformed circulating stem cells to the population of cells expressing Oct4 and Nanog we performed fluorescent in situ hybridization (FISH) with probes recognizing the *epidermal growth factor receptor* gene *(EGFR)*, which is amplified in roughly 40% of grade IV tumors [24]. Analysis of consecutive sections clearly demonstrated Oct4 and Nanog expression in tumor cells with EGFR gene amplification as well as in other tumor cells and other tumor samples without EGFR gene amplification (Fig. S7).

### Markers of all three germ-layers are represented in high grade gliomas

**Figure 4:**
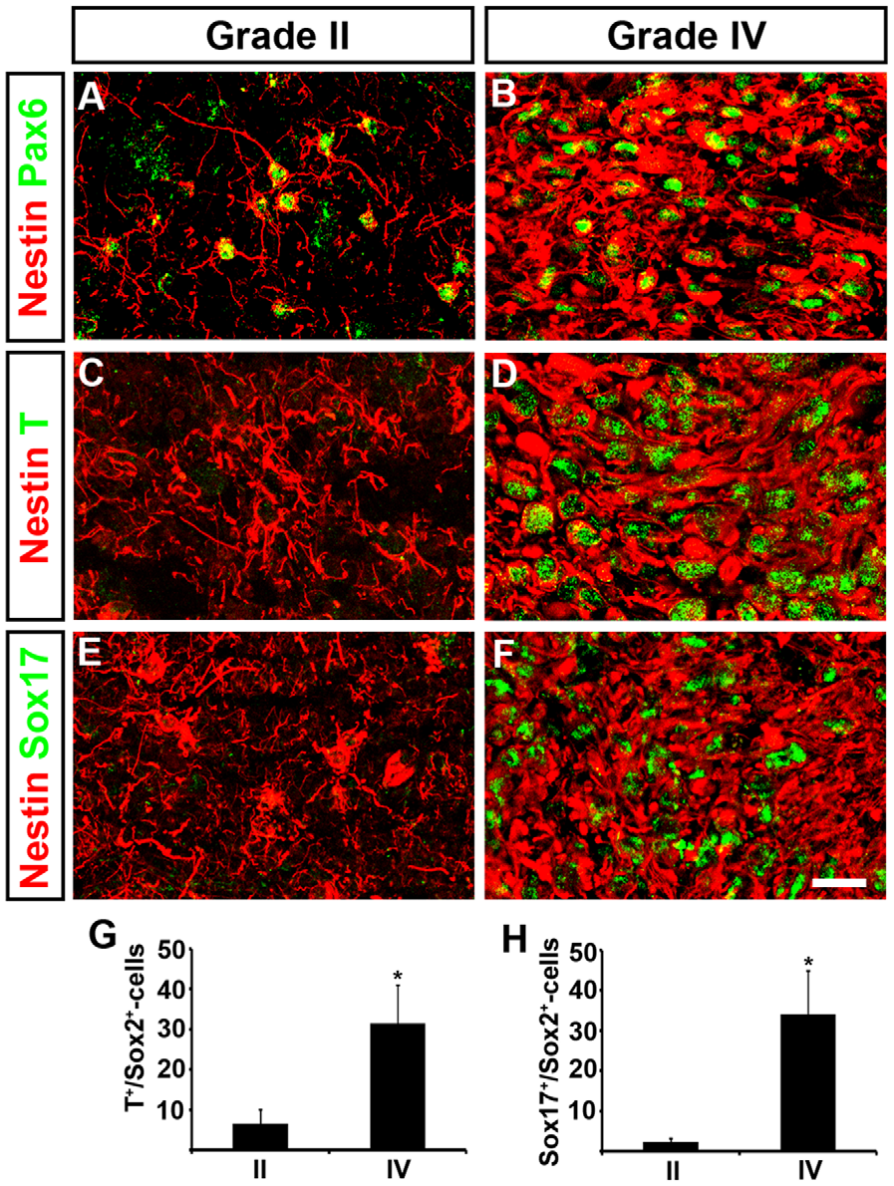
Increased malignancy is characterized by the acquisition of pluripotent properties. (A,B) Cells expressing the neural progenitor markers Pax6 and Nestin can be detected in both low (A) and high (B) grade glioma. (C–H) Expression of the mesodermal transcription factor T (C,D,G) and the endodermal marker Sox17 (E,F,H) are restricted to grade IV tumors. Scale bar; 20 µm in F. Data are represented as mean +/− SEM. * =  p<0.05, Student's t-test.

It has previously been demonstrated that cells of high grade gliomas are able to activate genetic programs leading to mesenchymal transformation and the suppression of neuronal- and glial-specific gene expression [25], but whether this finding reflects a pluripotent potential of glioma cells remains unresolved. To examine this possibility we analyzed tumor samples with an array of mesodermal and endodermal markers. Interestingly, apart from neural markers (Fig. 4A,B) many cells in grade IV tumors had also activated gene expression representative of the mesodermal lineage, including the brachyury homolog T [26], Dlx5 [27], Snail1 [28] and Myogenin [29] (Fig. 4C,D,G; Fig. S8). In addition, we could identify several endodermal lineage markers including Sox17 [30], [31], [32], Gata6 [33] and FoxA2 [34] (Fig. 4E,F,H; Fig. S8). Similar to the selective expression of Oct4 and Klf4 in high grade gliomas, mesodermal and endodermal markers were generally absent from grade II tumors (Fig. 4C,E,G,H). It is important to point out that none of the examined tumors expressed a full array of markers for mesodermal or endodermal cells, and in most cases cells co-expressed genes characteristic of different germ layers. For instance, T and Sox17 which define mesodermal and endodermal lineages respectively, could often be detected together with the NSC markers Sox1 and Nestin (Fig. 4D,F; and Fig. S8). Nevertheless, our data indicate that gene expression programs normally present in other than neural lineages are induced in high grade gliomas.

### Expression of pluripotent stem cell features depends on environmental signals

**Figure 5:**
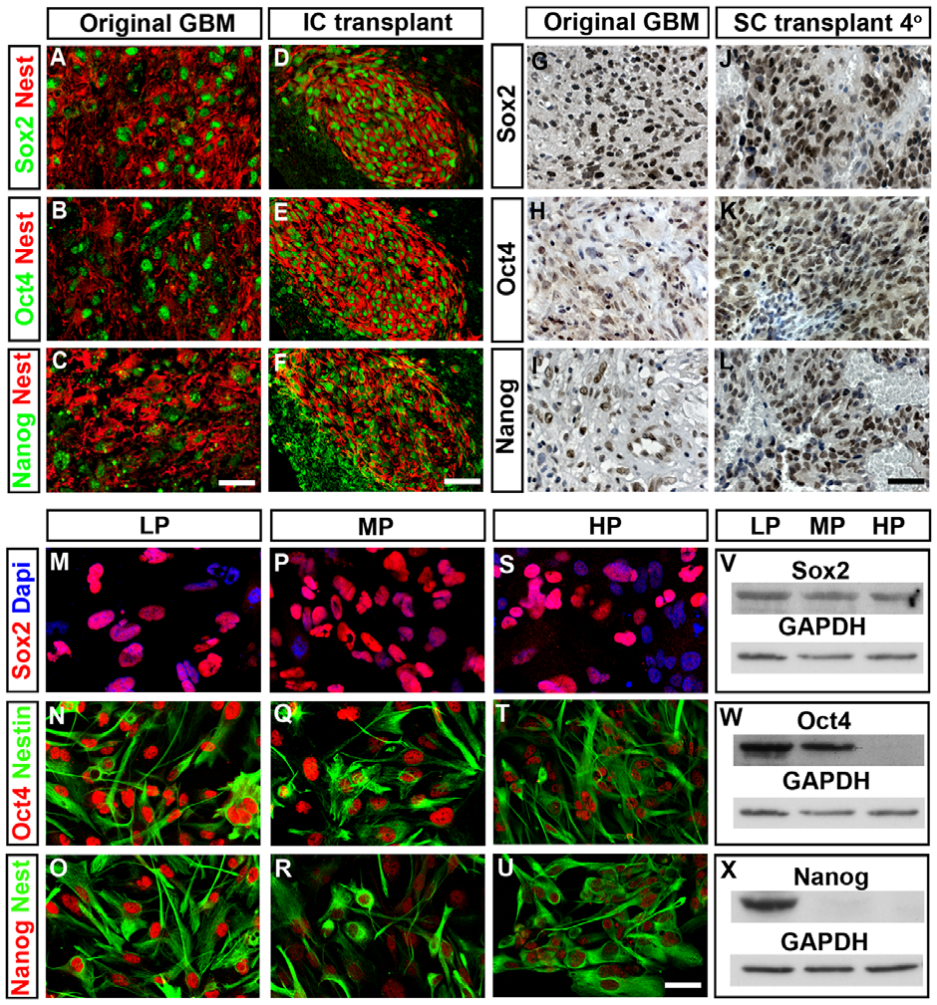
Stem cell features of high grade gliomas are dependent on the cellular niche. (A–F) The expression of Sox2 (A), Oct4 (B) and Nanog (C) was maintained in grade IV glioma cells subjected to intracranial engraftments in mice (D–F). The expression of Sox2 (G), Oct4 (H) and Nanog (I) was also maintained in cells exposed to repeated rounds of serial subcutaneous transplantations. Expression is here shown in quaternary (4°) tumors (J–L). (M–X) *In vitro* propagated tumor cells were examined for the expression of Sox2, Oct4 and Nanog protein expression, using cytohistochemistry and western blot analyses, after less than 10 passages (low passage, LP), between 10–20 passages (medium passage, MP) or after more than 20 passages (high passage, HP). While the expression of Sox2 was maintained for more than 50 passages (M,P,S,V) the expression of Oct4 (N,Q,T,W) and Nanog (O,R,U,X) declined after 10–20 passages. Quantification of three independent experiments show a significant loss of Oct4 (p<0.005) and Nanog (p<0.001) but not Sox2 (p>0.60) upon prolonged propagation, Student's t-test (Fig. S10). The loss of Oct4 and Nanog expression did not alter the expression of the neural progenitor marker Nestin (Q–R, T–U). *In vitro* studies and subcutaneous xenograft data are based on three distinct tumor samples. Intracranial xenograft data represent an additional tumor sample. Scale bars; 20 µm in C, 50 µm in F, 50 µm in L and 10 µm in U.

To better understand how environmental signals may influence the expression of stem cell properties, Sox2, Oct4 and Nanog expression was measured in primary human glioblastoma cells after intracranial engraftments, serial subcutaneous transplantations or *in vitro* culture. Resulting xenograft tumors, isolated two – three months after intracranial transplantations in mice, contained comparable amounts of Sox2^+^, Oct4^+^ and Nanog^+^ cells as the original human tumors (Fig. 5A–F and Fig. S9). Moreover, these markers could be detected in tumors generated after serial subcutaneous transplantations, and the number of Sox2^+^ cells expressing Oct4 and Nanog was essentially similar in primary and quaternary xenograft tumors (Fig. 5G–L; and data not shown). Glioma cells cultured *in vitro* initially expressed high levels of Sox2, Oct4 and Nanog (LP; low passage) (Fig. 5M–O,V–X). However, after a few passages their expression started to decline (Fig. 5Q,R,W,X) and after prolonged propagation (>20 passages) the expression of Oct4 and Nanog was generally lost (Fig. 5T,U,W,X and Fig. S10). In contrast, Sox2 expression remained unaltered and could be detected together with the NSC marker Nestin for more than 35 passages (Fig. 5S–V; Fig. S10 and data not shown). The expression of Oct4 and Nanog remained decreased also in cells cultured *in vitro* as spheres rather than in monolayer cultures (Fig. S11). Together, these data suggest that while NSC genes remain unaltered in glioma cells cultured *in vitro*, the expression of Oct4 and Nanog is dependent on extra-cellular regulatory cues present *in vivo* but not *in vitro*. It has been suggested that a hypoxic environment correlates with stem cells in glioblastomas [35], [36], [37], [38], [39]. To examine whether exposure to hypoxia increases the expression levels of stem cell markers we cultured low and high passage glioblastoma cells in normoxia (21% O_2_) and in hypoxia (1% O_2_). Although the cells exposed to hypoxia exhibited a robust upregulation of the Hypoxia Inducible Factor HIF1α, we could not detect any differences in Sox2, Oct4 or Nanog expression levels (Fig. S11). Neither could we detect any significant spatial correlation between cells expressing stem cell markers and neovascular niches as determined by the vasculature markers CD31 and CD105 (endoglin) (Fig. S11).

## Discussion

That several of the malignant features of glioblastomas, including aberrant growth, recurrence and radio-resistance, may be assigned cancer stem cells, argues that this cellular population constitutes a potential target for therapeutic strategies. However, to reach this goal it is necessary that we understand the mechanisms that promote stem cell maintenance in gliomas and endow cells with the capacity to initiate and sustain tumor growth. In this study we have demonstrated that glioblastomas express molecular similarities with both NSCs and pluripotent stem cells. For instance, SoxB1 proteins that in the healthy brain possess redundant and vital functions to maintain NSCs in an undifferentiated and proliferative state [40] are co-expressed in high grade gliomas but not in low grade. We also demonstrated that Sox3 had similar effect in NSCs as in glioma cells and and block cell cycle exit, whereas its dominant negative version promote cell cycle exit in both NSCs and glioma cells. In keeping with these findings, misexpression of Sox3 has previously been demonstrated to cause oncogenic transformation of embryonic fibroblasts [41] whereas microRNA-mediated suppression of Sox2 decreases the capacity of glioma cells to contribute to tumor growth [8]. Moreover, Sox3 was identified as a target of retroviral insertions causing T-cell lymphomas in mice [42] and the Sox2 locus is frequently amplified in prostate cancer [43]. Thus, several lines of evidence indicate that SoxB1 proteins are significant in promoting the undifferentiated state also in tumors.

Sox2, Oct4 and Nanog are vital for the development and maintenance of pluripotent stem cells [44], [45], [46], [47]. For instance, all three proteins have been assigned prominent roles in reprogramming somatic cells into iPS-cells [48] and loss of Nanog or Oct4 in ES-cells leads to the suppression of pluripotent stem cell features and transdifferentiation of ES-cells into extra-endodermal cells and trophectodermal cells, respectively [14], [44]. Moreover, gain-of-function experiments have demonstrated a role of Nanog and Oct4 in promoting cellular proliferation [49]. In this respect it is interesting that co-expression of Sox2, Oct4 and Nanog primarily could be detected in highly malignant forms of gliomas and that downstream target genes of these transcription factors in ES-cells, frequently are overexpressed in poorly differentiated tumors including glioblastomas [16]. Thus, it is possible that the transcription factors Sox2, Oct4 and Nanog that regulate self-renewal and pluripotency of stem cells have similar regulatory functions in gliomas. Indeed, suppression of Nanog expression in glioma cells reduced their tumorgenicity when transplanted into the mouse brain [50]For instance, their expression was accompanied by the presence of markers characteristic for the mesodermal or endodermal lineages. T protein, which is required for mesoderm formations [26], is significantly expressed in glioma tissue together with the muscle determination factor Myogenin [29]. In addition, we could detect expression of the endodermal regulators Sox17, Gata6 and FoxA2 [30], [31], [32], [33]. Although some of the endodermal and mesodermal proteins we identified, previously have been detected in association with the vasculature [51], [52], [53], the vast majority of the glioma cells expressing these markers were not associated with blood vessels in the tumors examined in this study. In contrast to neuronal and glial gene expression we were unable to detect cells expressing a complete array of mesodermal and endodermal markers. Instead, many cells expressing endodermal and mesodermal genes also expressed the neural progenitor cell markers Nestin and Sox1. Thus, rather than reflecting the formation of cells of endodermal or mesodermal lineages, it is conceivable that the mixed cellular phenotype in high grade gliomas results from deregulated gene expression, perhaps involving the activity of Sox2, Oct4 and Nanog.

One possible explanation for the differential expression of Sox1, Oct4 and Klf4 in low and high grade tumors, is that these tumor types may constitute distinct disease entities [54]. However, gene expression in cancer cells is also largely dependent on signals in the micro-environment. That signals in the micro-environment have regulatory roles in controlling cancer cell gene expression, is in agreement with our findings that cells isolated from high grade tumors rapidly downregulated the expression of Oct4 when cultured *in vitro*, while the expression was maintained in cells xenografted into the mouse brain or serially transplanted subcutaneously into mice. Thus, it is likely that regulators of pluripotency, such as Oct4, are selectively expressed in high grade gliomas as a result of a combinatorial activity of cell autonomous mechanisms and signals emanating from the tumor microenvironment.

In this paper we have demonstrated that glioblastomas display mixed characteristics of neural and pluripotent stem cells. Although our results indicate that the capacity of Sox3 to hinder differentiation is conserved between glioma cells and NSCs, further studies will be necessary to explain the exact role of Oct4, Nanog and Klf4 in gliomas. For instance, is their ability to promote the self-renewing and undifferentiated state conserved in gliomas and are they necessary for the pluripotent features expressed by glioblastomas? Nevertheless, it is tempting to propose that independently of the genetic alterations that may be present in cancer cells, stem cell regulatory programs may constitute vital targets for therapeutic innovations combating tumor growth.

## Materials and Methods

### Ethical statement and handling of human glioma samples

Twenty-three snap frozen human glioma samples were used in this work: 7 glioblastomas (WHO Grade IV), 6 WHO Grade III cases (2 anaplastic astrocytomas and 4 anaplastic oligoastrocytomas), 8 WHO Grade II cases (3 astrocytomas, 3 oligoastrocytomas, 2 oligodendrogliomas) and 2 WHO Grade I cases (2 pilocytic astrocytomas). Informed consent had been obtained from the patients before surgery (KI 02-254) and diagnosis was confirmed by a neuropathologist (AO). The snap frozen tissues used for qPCR had the following distribution: 30 glioblastomas (WHO Grade IV), 15 WHO Grade III cases (anaplastic astrocytomas), 19 WHO Grade II cases (astrocytomas) and 14 WHO Grade I cases (pilocytic astrocytomas). The samples used for qPCR were obtained according to the ethical guidelines approved by Department of Neurology, School of Medicine, University of Sao Paulo and by the Brazilian Health Ministry.

### Quantitative RT-PCR

Total RNA was extracted from snap frozen glioma samples with RNeasy Protect Mini Kit (Qiagen). First-strand cDNA was prepared using Superscript III (Invitrogen). qPCR was performed with SYBRgreen (Qiagen), using primers specific for human Oct4 and human Nanog. The housekeeping gene HPRT was used for cDNA normalization.

### Immunohistochemistry, immunofluorescence and *in situ* hybridization

Human glioma tissues and serial xenografted tissues were fixed in 4% paraformaldehyde (PFA) and embedded in paraffin. 5 µm sections were de-paraffinized and processed with antigen retrieval followed by antibody incubation at 4°C overnight. Stainings were visualized by DAB-based HRP-reaction and counterstaining performed with Haematoxylin. Snap frozen human tumor samples were cryosectioned. The 12 µm sections were fixed in 4% PFA for 15 minutes at room temperature. Sections were incubated overnight with primary antibodies at 4° Celsius. The antibodies used were: rabbit anti-Sox1 (1/1000, kind gift from Dr Wilson, Umeå University), goat anti-Sox2 (1/1000), guinea-pig anti-Sox3 (1/2000), rabbit anti-Sox2 (1/2000) (kindly provided by T. Edlund, Umeå University), mouse anti-Nestin (1/1000, Millipore Mab 5326), mouse anti-Ki67 (1/500, Abcam), rabbit anti-Ki67 (1/500, Neomarkers RB1510P0), rabbit anti-Neurofilament (1/1000, Serotec AHP286), rabbit anti-Oct4 (1/250, Santa Cruz sc9081), rabbit anti-Nanog (1/250, Abcam 21603), rabbit anti-Pax6 (1/500, Developmental Hybridoma Bank), goat anti-Brachyury (T) (1/250, Santa Cruz 17743), goat anti-Sox17 (1/250, Neuromics Gt15094), anti-Gata6 (1/500, Santa Cruz sc9055), mouse anti-FoxA2 (1/100, Developmental Hybridoma Bank), mouse anti-cd31 (1/400, Abcam ab9498), mouse anti-cd105 (1/400, DAKO M3527) and rabbit anti-Myogenin (1/250, Santa Cruz sc576). Images of stained sections were acquired with a Zeiss Axioplan Imager M1 mciroscope fitted with LSM5 confocal equipment. Images of cultured, fixed (4% PFA, 15 min, RT) cells and stained cells were acquired with a Zeiss Observer Z1 fluorescence microscope. The images were processed with Adobe Photoshop 4.0 software. *In situ* hybridization was performed as described [55] with probes specific for human *Sox1, Sox2* and *Sox3*.

### FISH

FISH was performed on formaldehyde-fixed 12- µm cryosections from patient-derived snap-frozen tumor samples using Vysis EGFR/CEP7 FISH probe Kit (Vysis, Abbott Laboratories) according to the manufacturer's protocol.

### Induction of hypoxia

Low and high passage glioma cells seeded in 100-mm dishes were incubated at 1% oxygen for 24 h in a Hypoxic Station (InVivo_2_ 300). Control cells were incubated under normoxic conditions (21% oxygen).

### Expression constructs and transfection

cDNAs were expressed in glioma cultures from the CMV-IE enhancer/chick β-actin promoter in the pCAGGS vector [56]. cDNAs encoding *Sox3-myc* and *Sox3-EnR* have been described elsewhere [9]. Expression vectors containing cDNAs encoding human *Sox1-myc, Sox2-myc, Sox3-myc, Oct4-myc* and *Nanog-myc* were obtained from Origene. DNA constructs were electroporated with Neon (Invitrogen) or transfected with Mirus TransIT (Mirus) into the cells.

### Western blot

The protein levels of Sox2, Oct4 and Nanog in different passages of glioma cells were assayed by Western blot. Cell lysates were prepared using modified RIPA buffer supplied with Protease Inhibitor Cocktail (Roche). Protein concentrations were determined using the BIO-Rad Protein Assay (Bio-Rad). Samples were separated in a 4–12% SDS-polyacrylamide gel (Invitrogen) according to standard procedures and blotted onto a PVDF membrane (Millipore). Immunoblotting was performed with commercially available antibodies (anti-Sox2 antibody, Chemicon; anti-Oct4 antibody, sc-5279, Santa Cruz; anti-Nanog antibody, eBioscience, anti-Hif1α, Bethyl A300-286A). ECL (Amersham) was applied for chemiluminescence detection. Immunoblotting signal quantifications were performed using the AIDA software, version 3.10.039 (Raytest, Straubenhardt, Germany)

### Serial subcutaneous xenograft assay and human glioma cell cultures

Human glioma tumor tissues were collected and pieces of the tissues were mechanically dissociated in a sterile Petri dish with a scalpel, followed by passage through syringe/needle with diminishing diameter. Two ml of cold PBS was used to collect the tumor tissue. A 0.6 mm needle was finally used to transfer 250 µl of minced tumor tissue subcutaneously to two SCID (Severe Combined Immune Deficiency) mice. Time to the development of visible tumor was registered. Tumor size was measured and monitored daily. When tumor size reached 500 mm^3^ the animals were sacrificed and tumors collected. Xenograft tumor tissues were collected and prepared the same way as original tissues, and applied for serial xenografts, (C207/1 Uppsala). At the time when the original human glioma tumor tissues were collected, parallel cell cultures were established, either maintained as monolayer with MEM media supplemented with 10% FBS or grown as spheres with neurosphere medium (#05751 Neurocult NA-S proliferation human kit; StemCell Technologies), supplemented with 20 ng/ml of EGF (Invitrogen), 20 ng/ml bFGF (Invitrogen) and 2 mg/ml Heparin (Sigma).

### Intracranial transplantation

Human tumor tissue was mechanically dissociated as described above. Depending on the amount of tumor tissue it was sometimes necessary to elute this with 0.5 to 1 ml of PBS. A 0.6 mm needle and Hamilton syringe with mechanical dispenser was finally used to transfer 10 µl of minced tumor tissue intracranially to each mouse. The injection was placed in the right frontal lobe of SCID mice. After two months or as soon as the mice showed any signs of illness, the animals were euthanized and brains were collected. The brains were fixed in PFA (2%) and then embedded in paraffin. Animal ethics committee approval no C159/98.

### Human ES cell culture and teratoma formation

The fully characterized permanent human embryonic stem cell (hESC) lines used as controls here, HS293 and HS346 have been derived from fresh poor quality embryos that had been donated for research after informed consent, and Karolinska Institutet Ethics Board approval, in the Fertility Unit of the Karolinska University Hospital, Huddinge, Sweden, as described earlier [57], [58]. They were derived using postnatal human skin fibroblasts as feeder cells and Knockout Serum Replacement (SR, Invitrogen, Scotland)-containing medium. For the present study, both lines were cultured feeder-free on Matrigel (Becton & Dickinson, San Jose, CA, USA) in mTeSR1 medium (StemCell Technologies, Grenoble, France) in small chambers for immunocytochemistry. The hESC lines have been karyotyped several times after derivation, and they were found repeatedly to be cytogenetically normal. After injection into testes of SCID mice, they have formed benign teratomas containing differentiated tissue components of the three germ layers. For teratoma formation, about 500,000 hESCs were harvested from culture plates, washed with PBS, and subsequently implanted beneath the testicular capsule of 7-week-old severe combined immunodeficiency (SCID/Beige) mice (C.B.-17/Gbms Tac-scid-bgDF N7, N&M, Ry, Denmark). Teratoma growth was determined by palpation every week, and the mice were euthanized (cervical dislocation) 8 weeks after implantation. The animals were housed in a specific pathogen-free facility of the Karolinska University Hospital Huddinge, in accordance with the ethics committee approval. The teratomas were fixed in formalin, and sections stained with haematoxylin and eosin to identify components of the germ layers. For the present study, sections for immunohistochemistry were plated on polylysine-coated object slides.

## Supporting Information

Figure S1 **Hematoxylin and eosin (H&E) analysis of grade IV glioma.** H&E characterizations demonstrate that the Sox1 mRNA in situ hybridization signal (A) overlaps with preserved tumor area in a grade IV sample with extensive necrotic and hemorrhagic areas (B). Image (C) represents magnification of framed regions in (A,B). Scale bars; 200 µm in B, 20 µm in C.
(TIF)

Figure S2 **Antibodies and in situ probes generated against Sox1, -2 or -3 are specific for their respective protein and mRNA.** (A–I) Antibodies against Sox1, Sox2 or Sox3 were used to stain 293HEK cells transfected with vectors expressing Myc-tagged versions of human Sox1 (A–C), Sox2 (D–F) or Sox3 (G–I). (J–L) Human embryonic stem cells express high amounts of Sox2 (K), but not Sox1 (J) or Sox3 (L). (M) Human Sox1, Sox2 or Sox3 cDNA was spotted onto Hybond-N membranes (1 pg–100 pg). The membranes were hybridized with DIG-labeled human Sox1, Sox2 or Sox3 RNA probes. Hybridization was detected with Nucleic Acid Detection kit for 1 hour. Scale bar: 20 µm in L.
(TIF)

Figure S3 **Antibodies generated against Oct4 and Nanog are specific for their respective protein.** (A–D) Antibodies, raised in mouse or rabbit, against Oct4 and Nanog were used to stain 293HEK cells transfected with vectors expressing Myc-tagged versions of human Oct4, (A,B) or Nanog (C,D). (E,F) Human embryonic stem cells express high amounts of Oct4 (E) and Nanog (F). (G,H) A substantial proportion of Tera2 human teratoma cells express Oct4 (G) and Nanog (H).Scale bar: 20 µm in H.
(TIF)

Figure S4 **Glioblastoma tissue expresses Nanog and Oct4.** Hematoxylin and eosin analyses demonstrate that high cellularity glioblastoma regions contain Nanog^+^ (A,B) and Oct4^+^ (C,D) cells. Scale bar: 20 µm in D.
(TIF)

Figure S5 **High level Klf4 expression is detected in high grade glioma.** The expression of Klf4 increased with increasing grades of malignancy. The amount of Sox2^+^ cells expressing Klf4 increased from approximately 17% in grade II tumors (A,C) to above 50% in grade IV gliomas (B,C). Data are represented as mean +/− SEM. *** = p<0.001, Student's t-test. Scale bar: 20 µm in B.
(TIF)

Figure S6 **Expression of pluripotent stem cell markers in human teratoma cells.** (A–E) In human ES derived teratoma xenografts the expression of Oct4 (D) and Nanog (E) is localized in areas with high Sox2 (B) expression but low Sox1 (A) and Sox3 (C) expression. (F) H&E staining of the region depicted in A–E (F). (G–J) Sox1 (G), Sox2 (H) and Sox3 (I) expression is high in rosette-like neurogenic regions with high levels of nestin (G) and tuj1 (H). These regions exhibit no expression of Oct4 (J) or Nanog (K). (L) H&E staining of the region depicted in G–K (L). Scale bar: 30 µm in L.
(TIF)

Figure S7 **Tumor cells expressing Oct4 and Nanog also harbor EGFR amplification on chromosome 7.** (A–F) Serial sections from two representative grade IV tumors showing (arrows in A, B) that the expression of Oct4 and Nanog is present in tumor areas with EGFR amplification. EGFR gene copies (red) are shown compared to the chromosome 7 specific probe (green) in the same nuclei. Scale bars: 10 µm in D and F.
(TIF)

Figure S8 **Mesodermal and endodermal markers present in glioblastoma.** (A–L) Grade IV tumors, but not grade II, H&E staining in (A,B) express the mesodermal markers Dlx5 (C,D), Snail (E,F), Myogenin (G,H) and the endodermal markers FoxA2 (I,J) and Gata6 (K,L). (M,N) Both the mesodermal marker T (M) and the endodermal marker Sox17 (N) could be detected together with the neural progenitor marker Sox1. Scale bar: 20 µm in N.
(TIF)

Figure S9 **Intracranially transplanted human glioma cells retain Oct4 expression and hypercellularity.** (A–C) H&E staining showing human glioma cells growing in normal mouse brain tissue (A). The boxed area is shown in higher magnification in (B,C). Intense combined Oct4 and Nestin staining is evident in the transplanted glioma cells but not in the surrounding tissue (C). Scale bar: 60 µm in C.
(TIF)

Figure S10 **Grade IV glioma cells exhibit progressive loss of Oct4 and Nanog but maintain Sox2 expression upon prolonged** ***in vitro*** **propagation.** (A–C) Sox2 protein levels are not significantly altered over repeated passages (A), whereas the levels of Oct 4 (B) and Nanog (C) protein are significantly lower after prolonged propagation. Data are represented as mean +/− SEM. ** = p<0.01, *** = p<0.001, Student's t-test, n = 3 for each condition and each marker.
(TIF)

Figure S11 **Expression of Sox2, Oct4 and Nanog in tumors is not restricted to the vascular niche. Sox2, Oct4 and Nanog expression is not induced by hypoxia or by growing late passage cells as spheres.** (A–C) Low passage cells grown as gliospheres express Sox2 (A), Oct4 (B), Nanog (C) and Nestin (A–C). (D–F) High passage cells maintain Sox2 (D) and Nestin (D–F) expression but do not express Oct4 (E) or Nanog (F). (G) The change from normoxic conditions (21%) to hypoxic conditions (1%) robustly induces Hif1α expression but does not affect expression levels of Sox2, Oct4 or Nanog neither in cells from low passage nor in high passage cells. (H–M) Serial sections (H–J, K–M) from primary GBM tumors showing that expression of Sox2, Oct4 and Nanog is not restricted to the vascular niche as outlined by the expression of CD31 (H–J) and CD105 (K–M). Scale bars: 50 µm in F and 60 µm in M.
(TIF)
