# Supplementary figures and images for: Activation of Neural and Pluripotent Stem Cell Signatures Correlates with Increased Malignancy in Human Glioma
Source: PLoS One. 2011 Mar 31;6(3):e18454. doi: 10.1371/journal.pone.0018454 (PMC3069091; doi:10.1371/journal.pone.0018454)

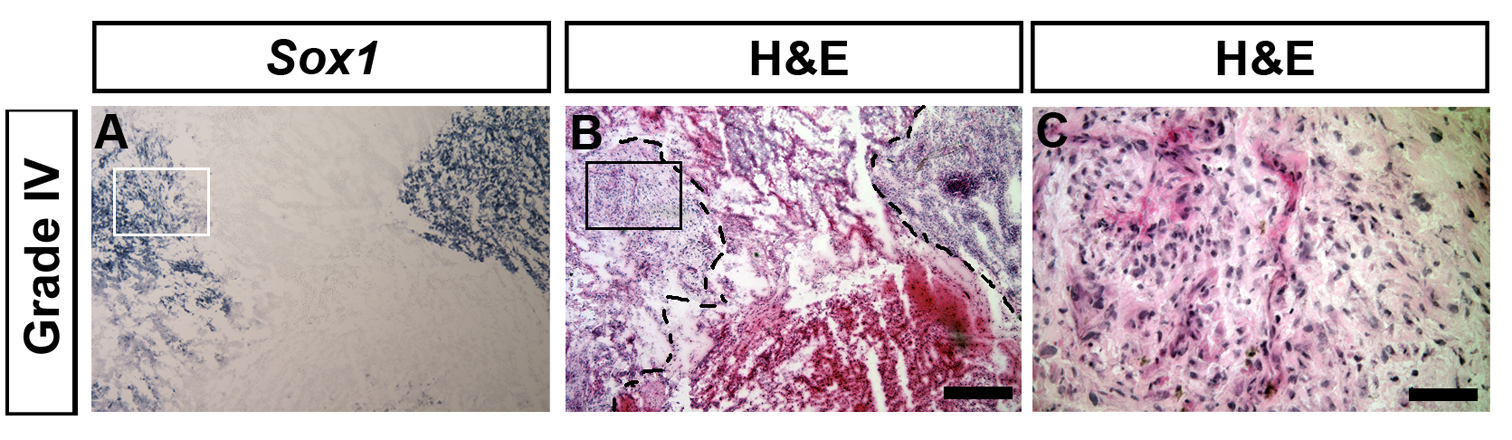

Supplement: Figure S1 — Hematoxylin and eosin (H&E) analysis of grade IV glioma. H&E characterizations demonstrate that the Sox1 mRNA in situ hybridization signal (A) overlaps with preserved tumor area in a grade IV sample with extensive necrotic and hemorrhagic areas (B). Image (C) represents magnification of framed regions in (A,B). Scale bars; 200 µm in B, 20 µm in C. (TIF) [file pone.0018454.s001.tif]

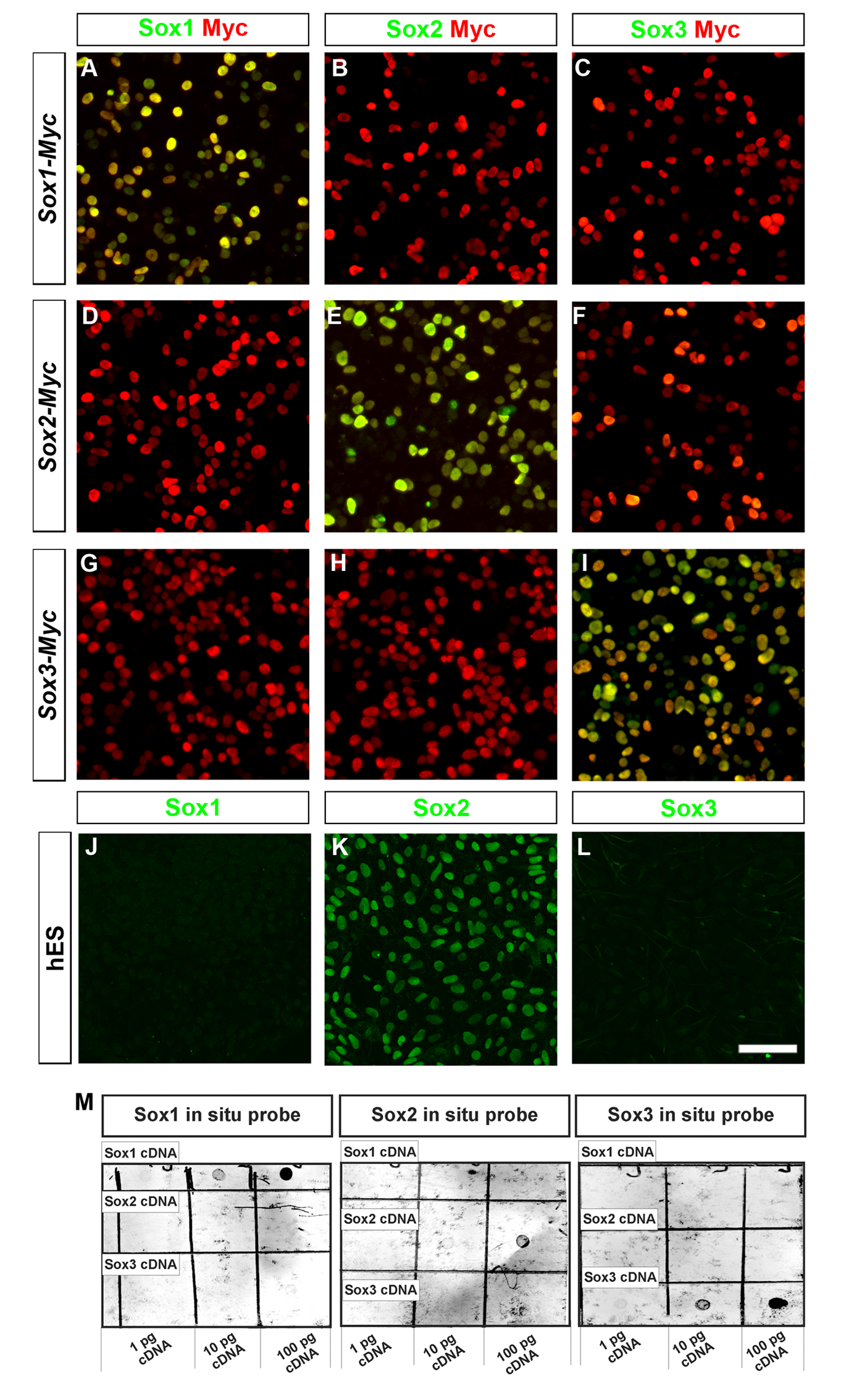

Supplement: Figure S2 — Antibodies and in situ probes generated against Sox1, -2 or -3 are specific for their respective protein and mRNA. (A–I) Antibodies against Sox1, Sox2 or Sox3 were used to stain 293HEK cells transfected with vectors expressing Myc-tagged versions of human Sox1 (A–C), Sox2 (D–F) or Sox3 (G–I). (J–L) Human embryonic stem cells express high amounts of Sox2 (K), but not Sox1 (J) or Sox3 (L). (M) Human Sox1, Sox2 or Sox3 cDNA was spotted onto Hybond-N membranes (1 pg–100 pg). The membranes were hybridized with DIG-labeled human Sox1, Sox2 or Sox3 RNA probes. Hybridization was detected with Nucleic Acid Detection kit for 1 hour. Scale bar: 20 µm in L. (TIF) [file pone.0018454.s002.tif]

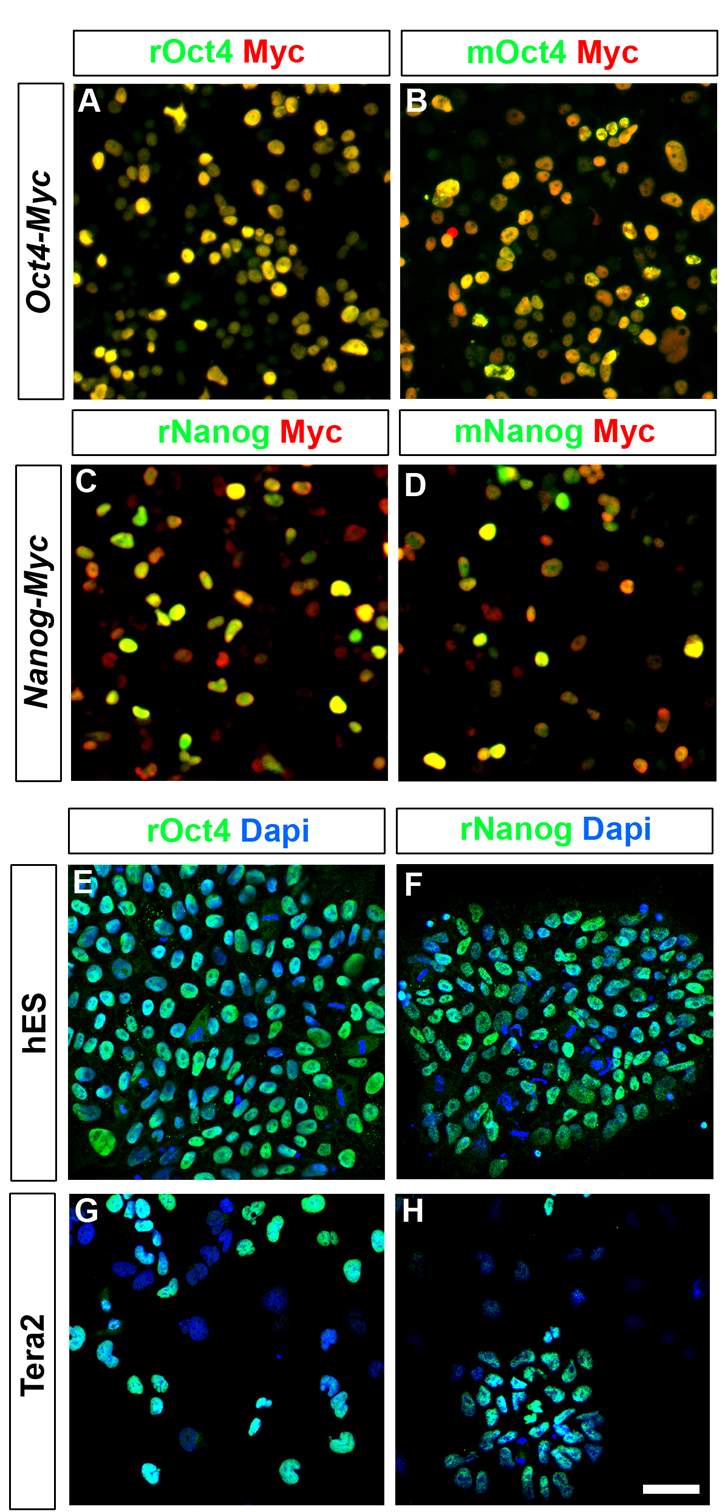

Supplement: Figure S3 — Antibodies generated against Oct4 and Nanog are specific for their respective protein. (A–D) Antibodies, raised in mouse or rabbit, against Oct4 and Nanog were used to stain 293HEK cells transfected with vectors expressing Myc-tagged versions of human Oct4, (A,B) or Nanog (C,D). (E,F) Human embryonic stem cells express high amounts of Oct4 (E) and Nanog (F). (G,H) A substantial proportion of Tera2 human teratoma cells express Oct4 (G) and Nanog (H).Scale bar: 20 µm in H. (TIF) [file pone.0018454.s003.tif]

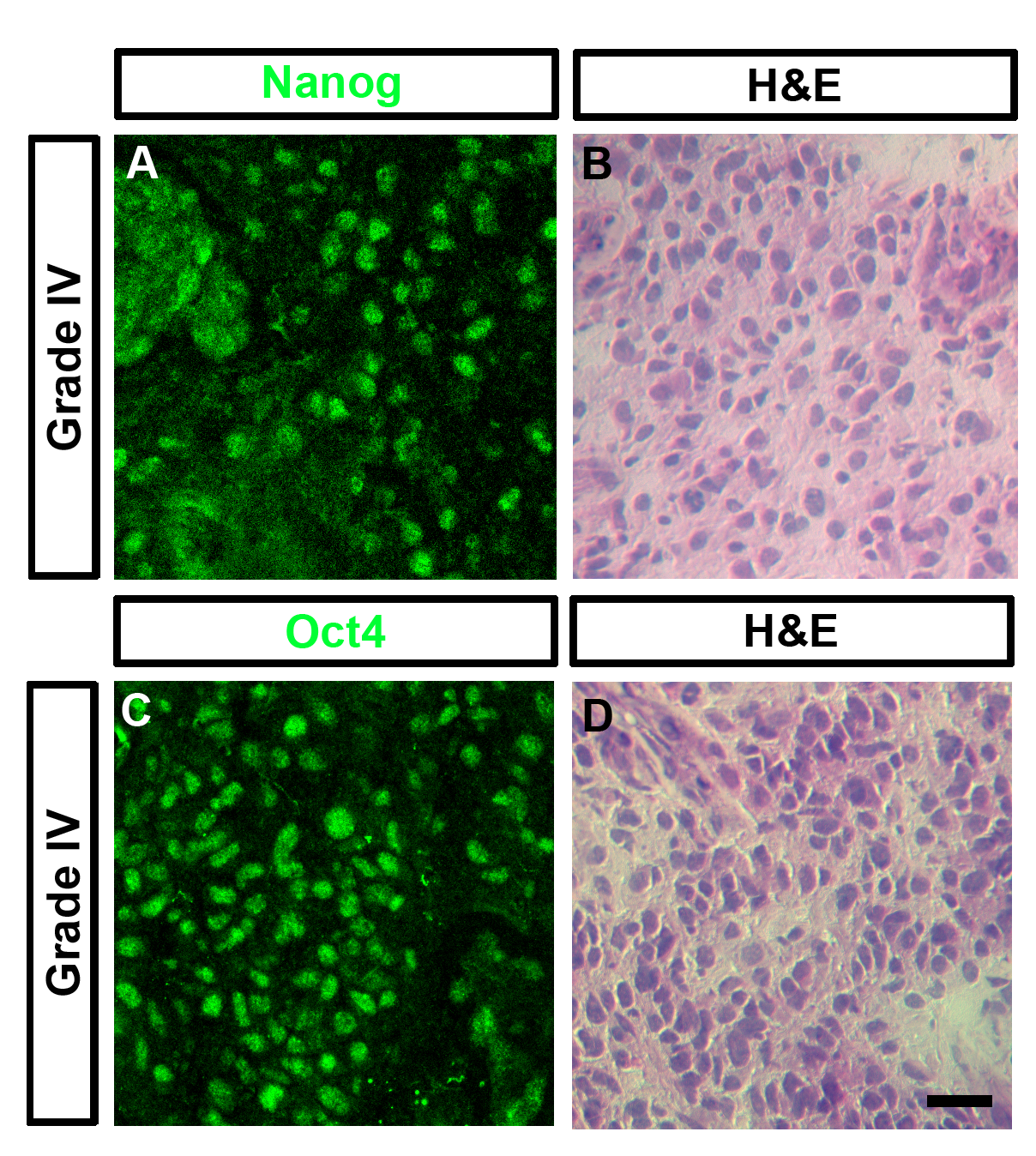

Supplement: Figure S4 — Glioblastoma tissue expresses Nanog and Oct4. Hematoxylin and eosin analyses demonstrate that high cellularity glioblastoma regions contain Nanog+ (A,B) and Oct4+ (C,D) cells. Scale bar: 20 µm in D. (TIF) [file pone.0018454.s004.tif]

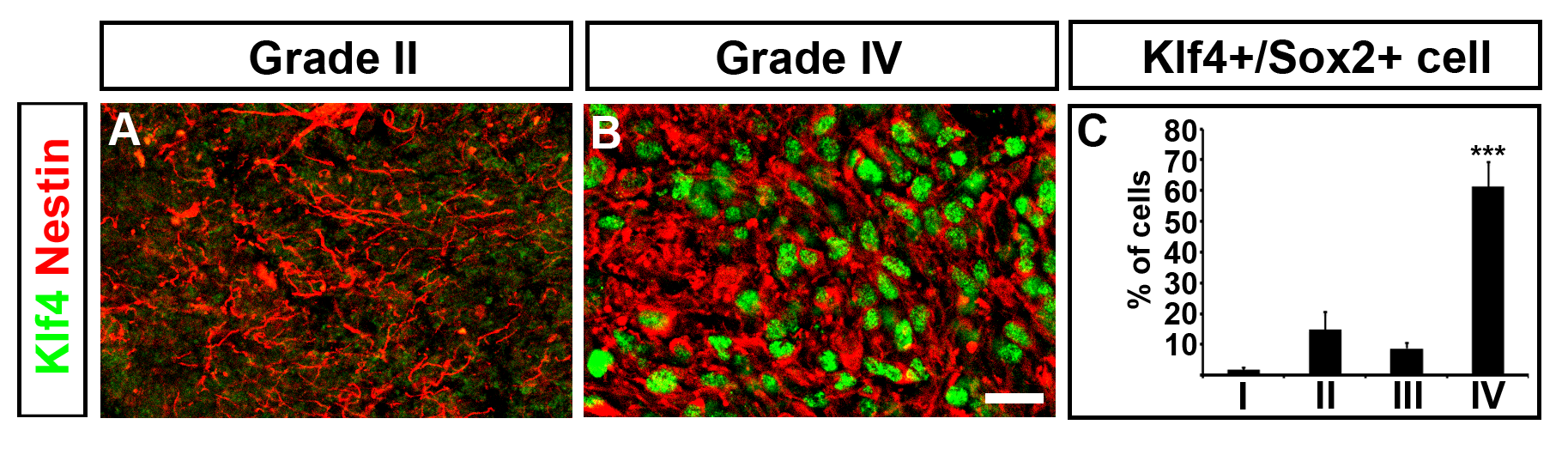

Supplement: Figure S5 — High level Klf4 expression is detected in high grade glioma. The expression of Klf4 increased with increasing grades of malignancy. The amount of Sox2+ cells expressing Klf4 increased from approximately 17% in grade II tumors (A,C) to above 50% in grade IV gliomas (B,C). Data are represented as mean +/− SEM. *** = p<0.001, Student's t-test. Scale bar: 20 µm in B. (TIF) [file pone.0018454.s005.tif]

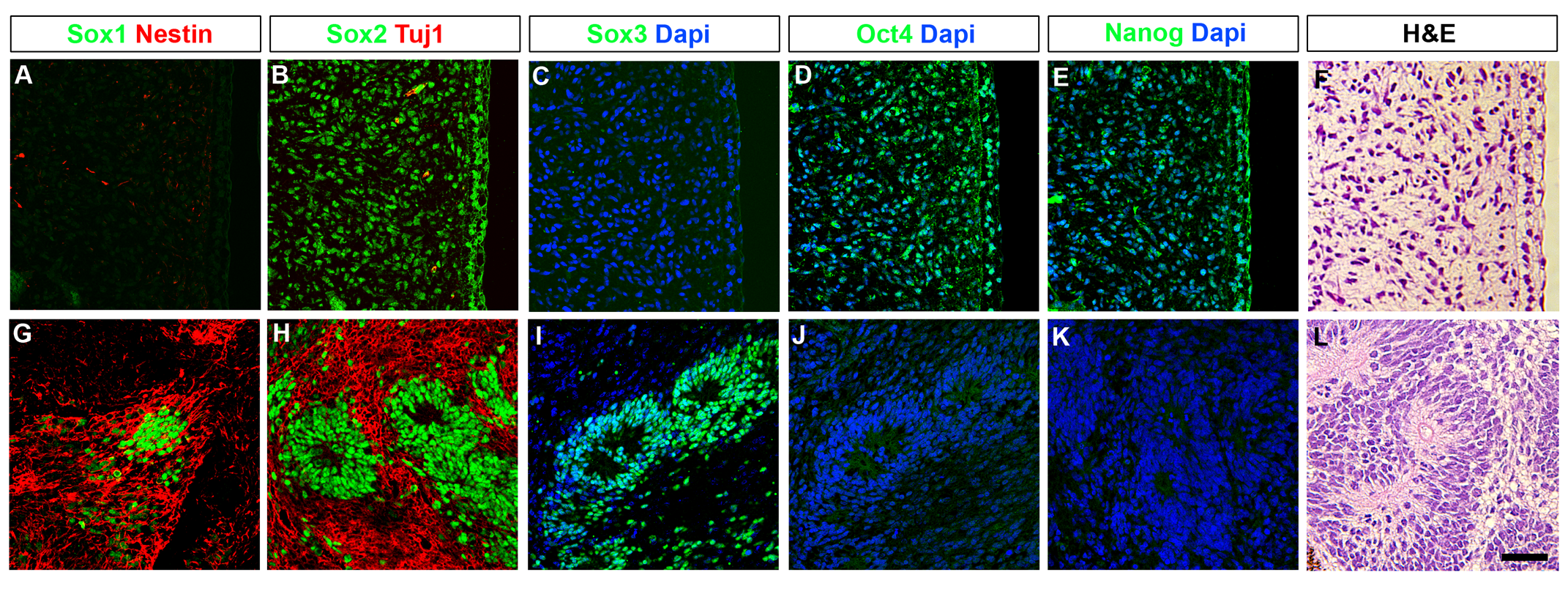

Supplement: Figure S6 — Expression of pluripotent stem cell markers in human teratoma cells. (A–E) In human ES derived teratoma xenografts the expression of Oct4 (D) and Nanog (E) is localized in areas with high Sox2 (B) expression but low Sox1 (A) and Sox3 (C) expression. (F) H&E staining of the region depicted in A–E (F). (G–J) Sox1 (G), Sox2 (H) and Sox3 (I) expression is high in rosette-like neurogenic regions with high levels of nestin (G) and tuj1 (H). These regions exhibit no expression of Oct4 (J) or Nanog (K). (L) H&E staining of the region depicted in G–K (L). Scale bar: 30 µm in L. (TIF) [file pone.0018454.s006.tif]

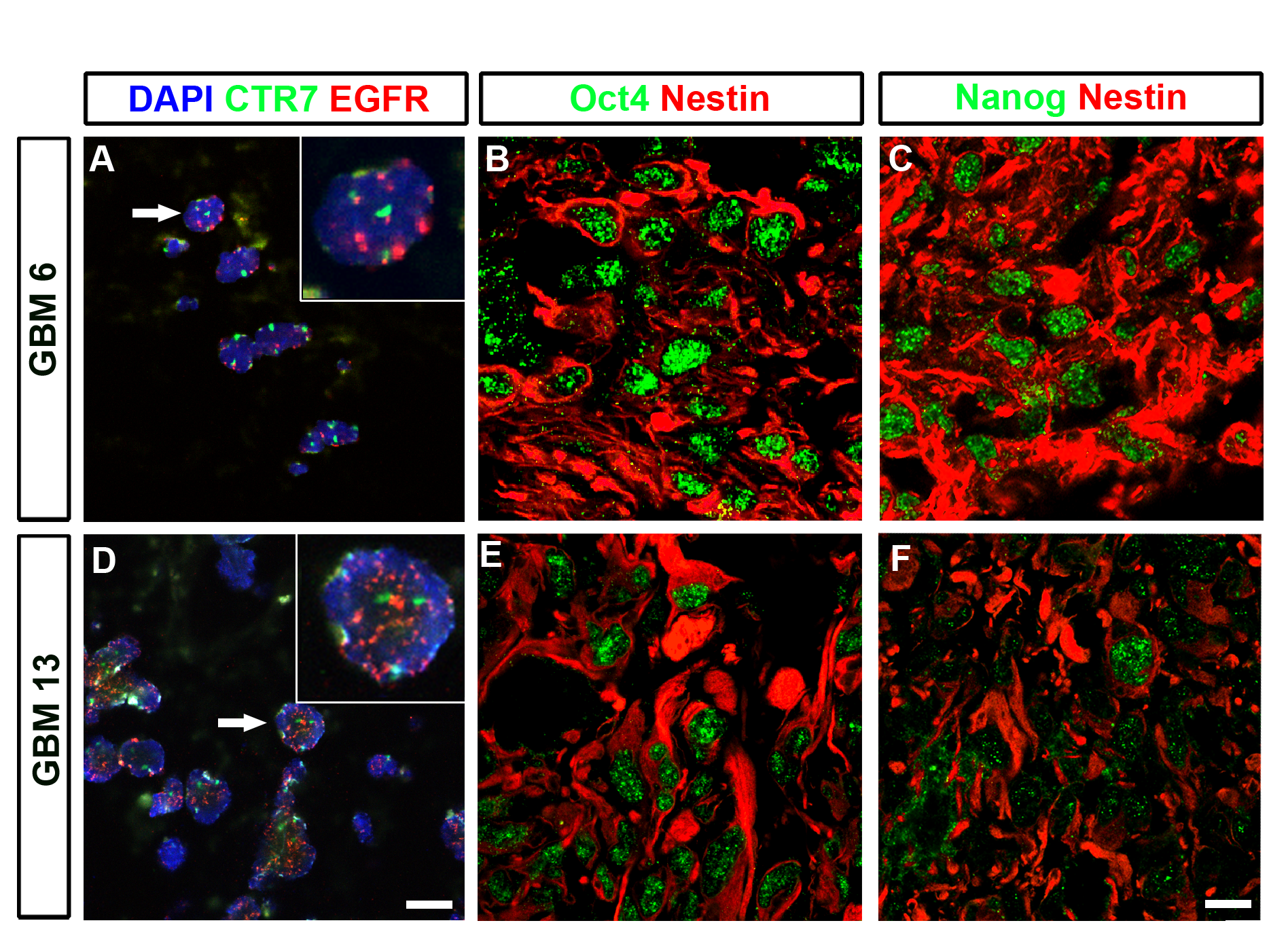

Supplement: Figure S7 — Tumor cells expressing Oct4 and Nanog also harbor EGFR amplification on chromosome 7. (A–F) Serial sections from two representative grade IV tumors showing (arrows in A, B) that the expression of Oct4 and Nanog is present in tumor areas with EGFR amplification. EGFR gene copies (red) are shown compared to the chromosome 7 specific probe (green) in the same nuclei. Scale bars: 10 µm in D and F. (TIF) [file pone.0018454.s007.tif]

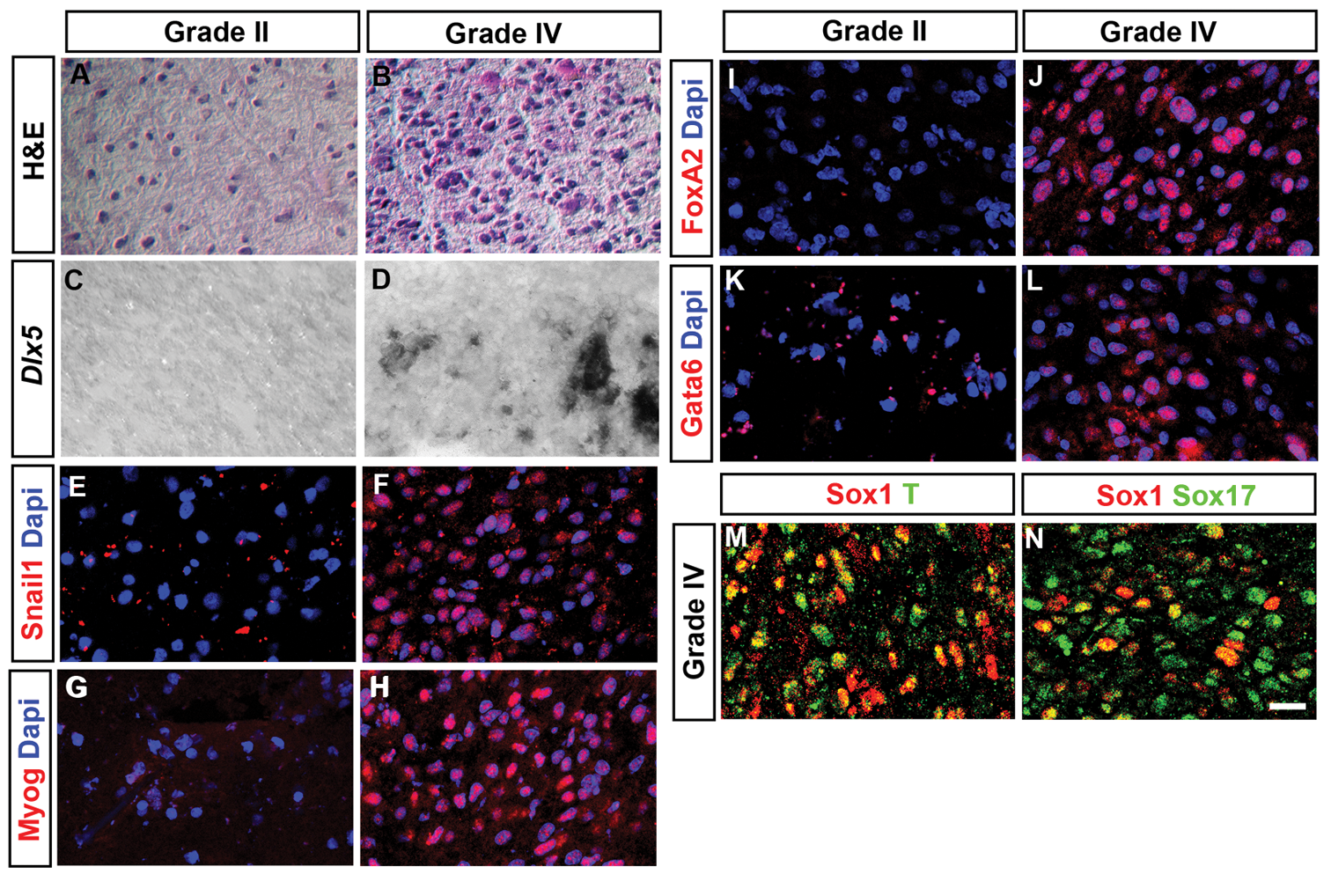

Supplement: Figure S8 — Mesodermal and endodermal markers present in glioblastoma. (A–L) Grade IV tumors, but not grade II, H&E staining in (A,B) express the mesodermal markers Dlx5 (C,D), Snail (E,F), Myogenin (G,H) and the endodermal markers FoxA2 (I,J) and Gata6 (K,L). (M,N) Both the mesodermal marker T (M) and the endodermal marker Sox17 (N) could be detected together with the neural progenitor marker Sox1. Scale bar: 20 µm in N. (TIF) [file pone.0018454.s008.tif]

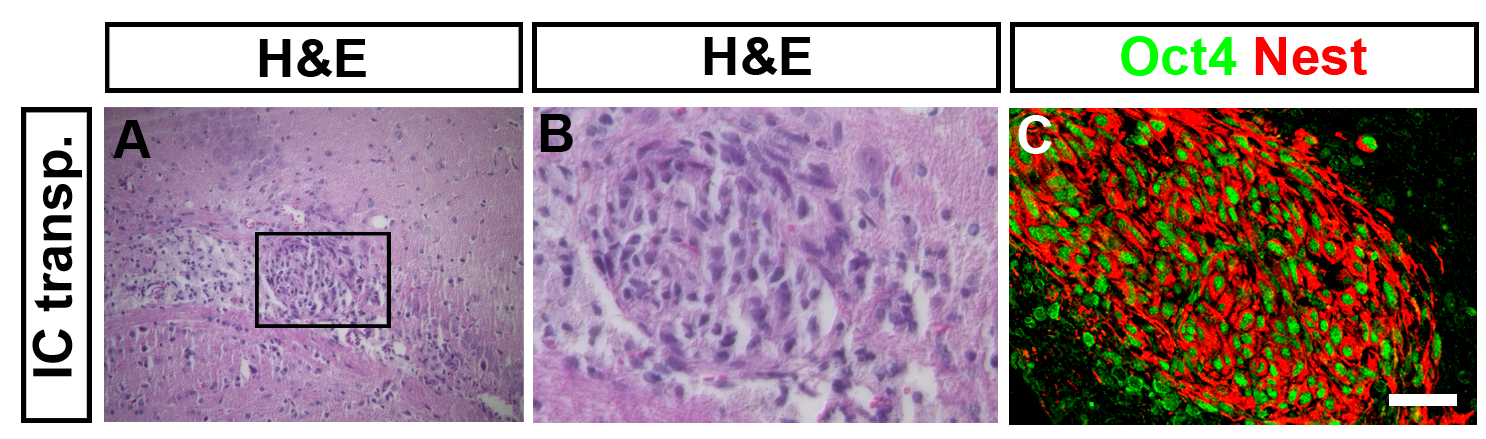

Supplement: Figure S9 — Intracranially transplanted human glioma cells retain Oct4 expression and hypercellularity. (A–C) H&E staining showing human glioma cells growing in normal mouse brain tissue (A). The boxed area is shown in higher magnification in (B,C). Intense combined Oct4 and Nestin staining is evident in the transplanted glioma cells but not in the surrounding tissue (C). Scale bar: 60 µm in C. (TIF) [file pone.0018454.s009.tif]

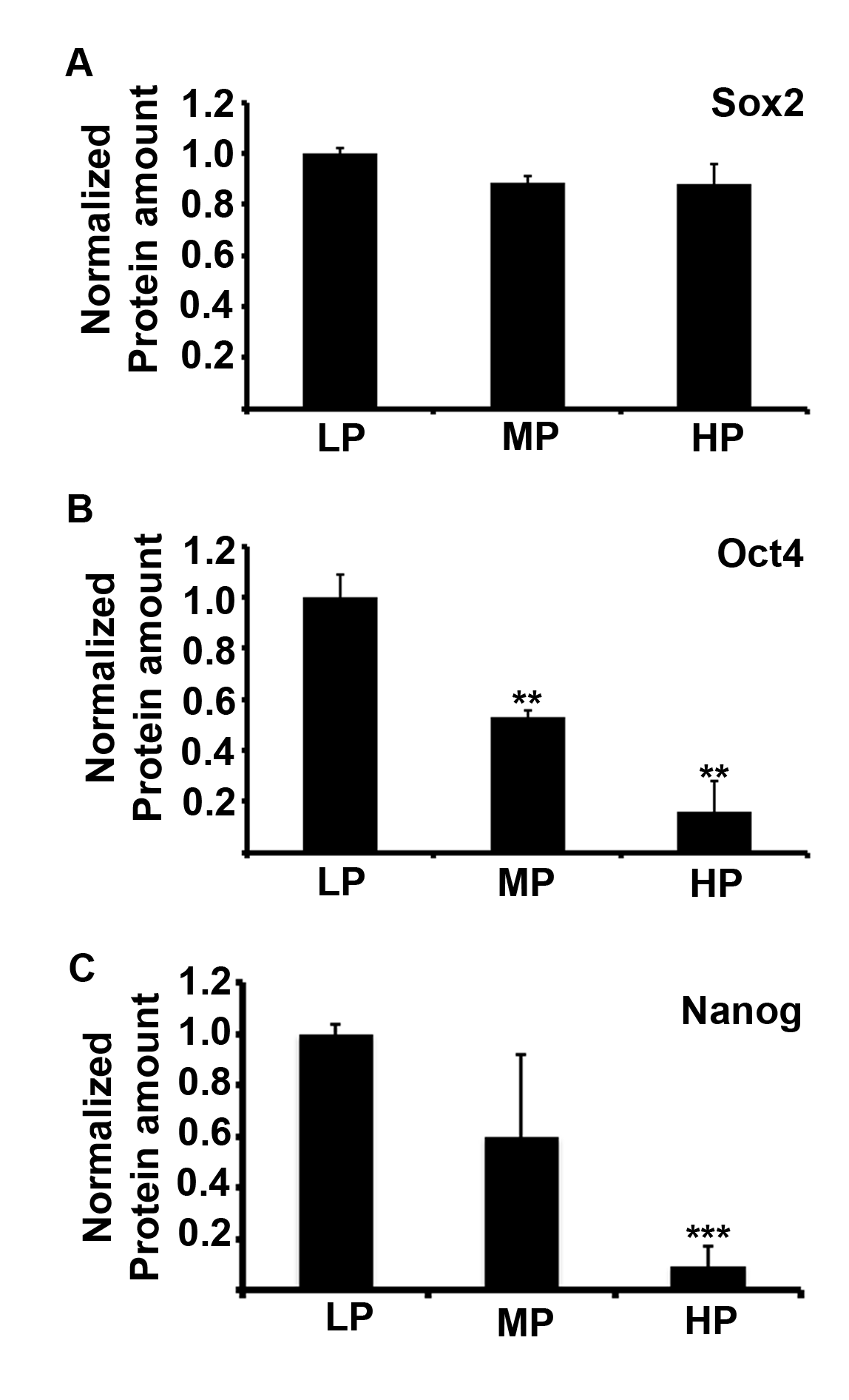

Supplement: Figure S10 — Grade IV glioma cells exhibit progressive loss of Oct4 and Nanog but maintain Sox2 expression upon prolonged in vitro propagation. (A–C) Sox2 protein levels are not significantly altered over repeated passages (A), whereas the levels of Oct 4 (B) and Nanog (C) protein are significantly lower after prolonged propagation. Data are represented as mean +/− SEM. ** = p<0.01, *** = p<0.001, Student's t-test, n = 3 for each condition and each marker. (TIF) [file pone.0018454.s010.tif]

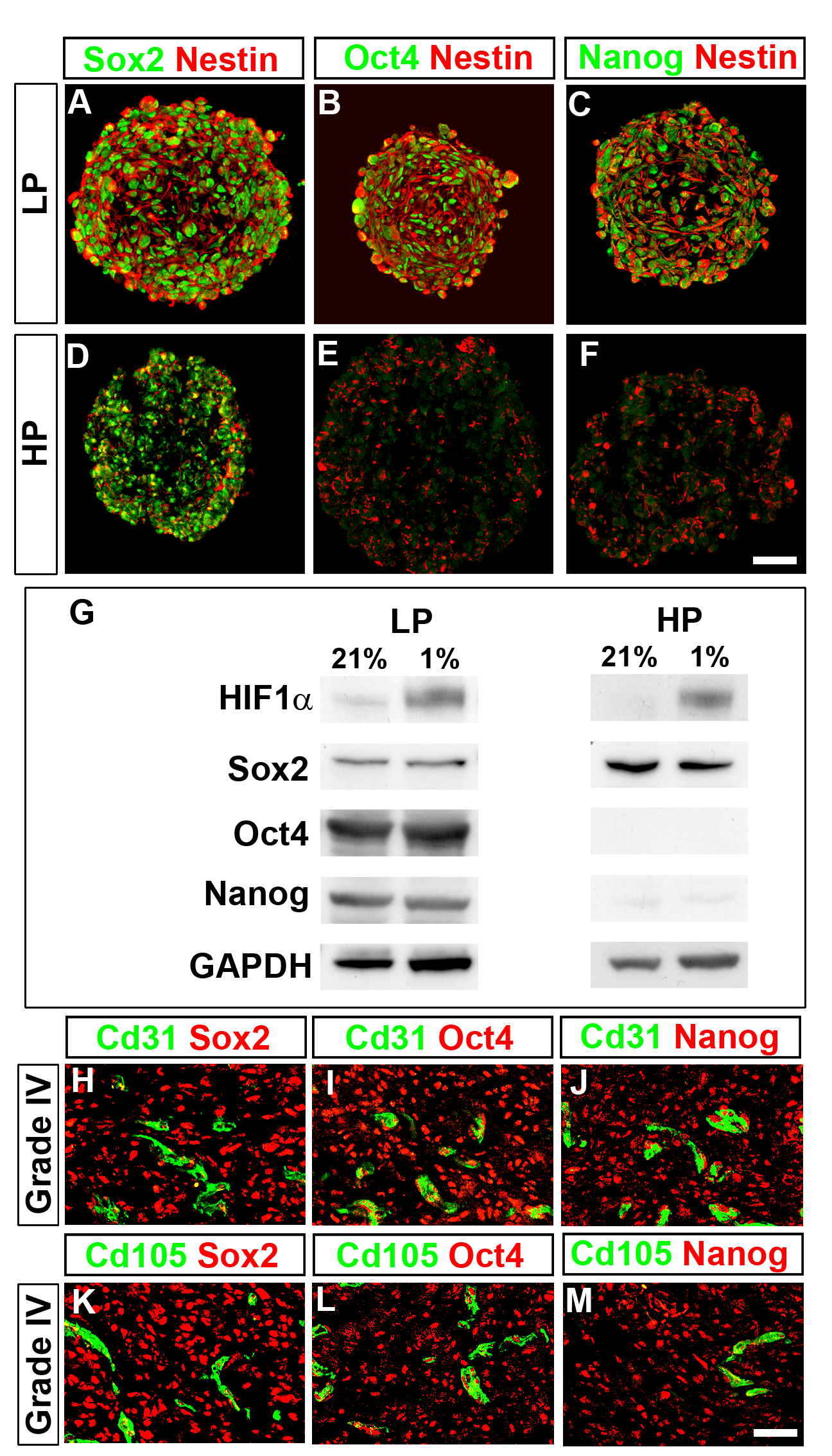

Supplement: Figure S11 — Expression of Sox2, Oct4 and Nanog in tumors is not restricted to the vascular niche. Sox2, Oct4 and Nanog expression is not induced by hypoxia or by growing late passage cells as spheres. (A–C) Low passage cells grown as gliospheres express Sox2 (A), Oct4 (B), Nanog (C) and Nestin (A–C). (D–F) High passage cells maintain Sox2 (D) and Nestin (D–F) expression but do not express Oct4 (E) or Nanog (F). (G) The change from normoxic conditions (21%) to hypoxic conditions (1%) robustly induces Hif1α expression but does not affect expression levels of Sox2, Oct4 or Nanog neither in cells from low passage nor in high passage cells. (H–M) Serial sections (H–J, K–M) from primary GBM tumors showing that expression of Sox2, Oct4 and Nanog is not restricted to the vascular niche as outlined by the expression of CD31 (H–J) and CD105 (K–M). Scale bars: 50 µm in F and 60 µm in M. (TIF) [file pone.0018454.s011.tif]
